# Pseudo-Meigs syndrome: à propos d'un cas

**DOI:** 10.11604/pamj.2014.17.184.3875

**Published:** 2014-03-10

**Authors:** Benjelloun Hanane, Morad Sanaa, Zaghba Nahid, Bakhatar Abdelaziz, Yassine Najiba, Bahlaoui Abdelkrim

**Affiliations:** 1 Service des Maladies Respiratoires, Centre hospitalier Ibn Rochd, Casablanca, Maroc

**Keywords:** Pseudo-Meigs syndrome, cancer, ovaire, épanchements, CA 125, Pseudo-Meigs syndrome, cancer, ovary, effusion, CA 125

## Abstract

**Introduction:**

Le pseudo-Meigs syndrome comprend une tumeur pelvienne bénigne (en dehors des fibrothécomes ovariens) ou maligne associée à des épanchements, pleural et ou péritonéal, qui disparaissent après l'exérèse tumorale. Nous rapportons l'observation d'une patiente âgée de 47 ans, qui présentait une pleurésie droite associée à une ascite et une tumeur ovarienne maligne. L’évolution après l'exérèse chirurgicale de la masse tumorale même en l'absence d'une chimiothérapie montrait une disparition des épanchements. Le pseudo-Meigs syndrome est rare. Il nécessite des biopsies pleurales et péritonéales négatives, et la disparition des épanchements après l'exérèse chirurgicale de la tumeur, avant toute chimiothérapie. Une telle distinction est importante aussi bien sur le plan thérapeutique que pronostique.

## Introduction

Le pseudo-Meigs syndrome est une triade comportant une tumeur pelvienne maligne ou bénigne (en dehors des fibro-thécomes ovariens) associée à des épanchements, pleural et péritonéal, qui disparaissent après l'exérèse tumorale [1]. Attribuer ces épanchements pleural et péritonéal, au cours d'un cancer de l'ovaire, à ce syndrome est une situation exceptionnelle qui nécessite des preuves histologiques et surtout évolutives. Nous rapportons un cas au cours d'un cystadénocarcinome ovarien de bonne évolution après l'exérèse chirurgicale et avant de démarrer la chimiothérapie.

## Patient et observation

**Figure 1:**
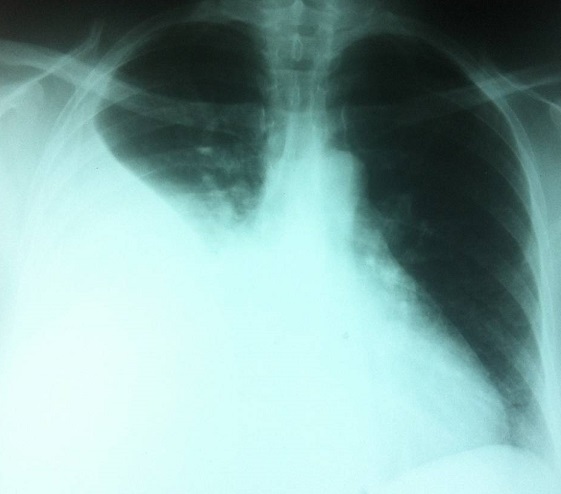
Radio du thorax montrant une opacité de type pleural occupant les deux tiers inférieurs de l'hémithorax droit

**Figure 2:**
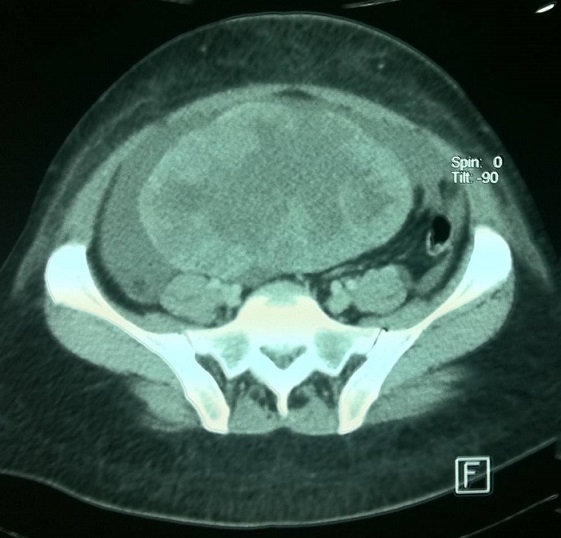
Coupe tomodensitométrique pelvienne montrant une masse ovarienne droite solidokystique qui mesurait 143 x 114 x 83 mm

**Figure 3:**
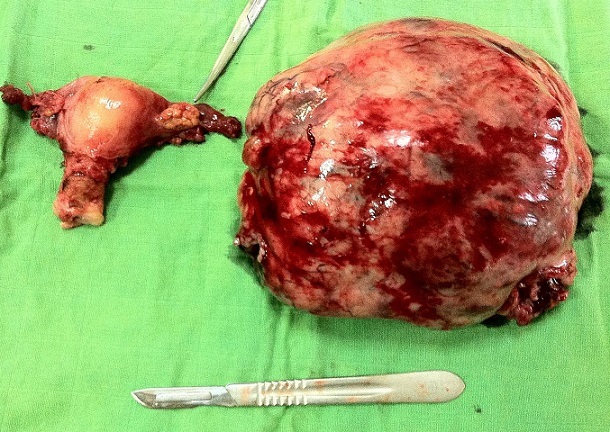
Exploration chirurgicale montrant un ovaire droit siège d'une tumeur de 16x14 centimètres

**Figure 4:**
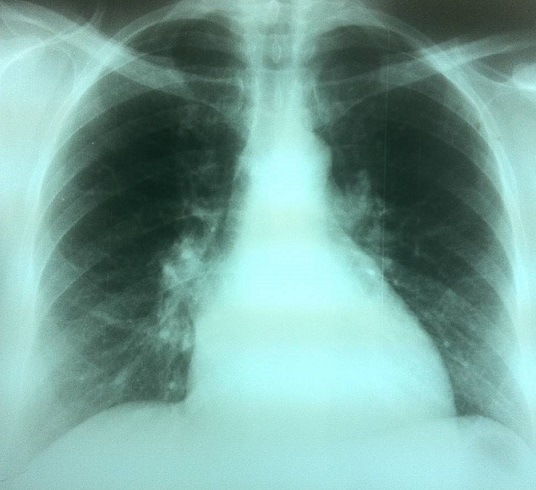
Radio du thorax réalisée deux mois après la chirurgie montrant un assèchement total de la pleurésie droite

Une patiente âgée de 47 ans, en périménopause, sans antécédents pathologiques particuliers. Elle consultait pour une distension abdominale associée à une sensation de pesanteur pelvienne, une douleur basithoracique droite à type de point de côté, une toux sèche et une dyspnée d'effort. Le tout évoluait dans un contexte de fléchissement l’état général. L'examen clinique retrouvait un syndrome d’épanchement liquidien des deux tiers inférieurs de l'hémithorax droit, une distension abdominale avec une matité diffuse et un signe de flot positif, une masse abdomino-pelvienne qui mesurait douze centimètres de diamètre, ferme, latéralisée à droite par rapport à l'utérus, avec un signe de glaçon positif. Le téléthorax montrait une opacité de type pleural occupant les deux tiers inferieurs de l'hémithorax droit (Figure 1). La ponction pleurale montrait un liquide jaune citrin exsudatif, lymphocytaire. Trois ponctions biopsies pleurales montraient un remaniement inflammatoire chronique non spécifique, sans signes de malignité. L’étude cytodiagnostique du liquide pleural n'avait pas montré de cellules malignes. La pleurésie récidivait après plusieurs ponctions évacuatrices. L’échographie abdominopelvienne montrait une ascite de grande abondance anéchogène et libre, une masse solidokystique latéro-utérine droite, à priori ovarienne, mesurant 14 x 12 centimètres. La tomodensitométrie thoraco-abdomino-pelvienne montrait une masse ovarienne droite solidokystique qui mesurait 143 x 114 x 83 mm (Figure 2), des épanchements pleural droit et abdominal de grande abondance. Il n'existait pas de lésions parenchymateuses pulmonaires, ni d'adénopathies médiastinales, iliaques ou lombo-aortiques. Le dosage sérique du CA-125 était élevé à 161 UI/mL pour une normale inférieure à 35 UI/mL. Une laparotomie diagnostique et thérapeutique était indiquée. L'exploration chirurgicale retrouvait une ascite de grande abondance de trois litres qui était aspirée, un ovaire droit siège d'une tumeur de 16x14 centimètres (Figure 3), Il n'existait pas de nodules péritonéaux, épiploïques ou hépatiques, ni d'adénopathies pelviennes ou lomboaortiques palpables. Une biopsie tumorale était examinée histologiquement en extemporanée objectivant un processus tumoral malin. Une hystérectomie totale sans conservation annexielle, un curage ganglionnaire pelvien et lombo-aortique, une omentectomie, des biopsies péritonéales et pariétales et un prélèvement du liquide d'ascite étaient réalisés. L'examen anatomopathologique de la pièce opératoire montrait un cystadénocarcinome séreux, unilatéral de l'ovaire droit essentiellement solide avec effraction capsulaire. L'ovaire controlatéral était normal. Il n'existait pas de métastase ganglionnaire, omentales, péritonéales ou pariétales. Le cytodiagnostic du liquide d'ascite ne montrait pas de cellules malignes. Le cancer ovarien était classée stade Ic de la FIGO (fédération internationale de gynécologie-obstétrique). Les suites opératoires étaient simples. Des contrôles cliniques, radiologiques (Figure 4) et échographiques réalisés une semaine, deux semaines, un mois et deux mois après l'intervention et avant de démarrer la chimiothérapie ne montraient pas d’épanchement pleural ni péritonéal. La régression de la pleurésie et l'ascite était spectaculaire, immédiatement après l'exérèse chirurgicale et en absence de toute chimiothérapie. La patiente n'avait pas reçu de chimiothérapie par refus de celle-ci. Des contrôles sériques du CA 125, réalisés dix jours puis un mois après l'intervention, étaient respectivement à 16 et 12 UI/mL. Avec un recul de douze mois, aucune récidive n’était notée.

## Discussion

Le syndrome de Demons-Meigs comprend une tumeur ovarienne bénigne, associée à une ascite et une pleurésie le plus souvent droite, dont l'exérèse entraine la disparition des épanchements. Il est le plus souvent décrit avec les fibrothécomes ou les tumeurs de la granulosa. En 1884, Albert Demons, médecin français, décrit l'association d'un kyste ovarien à un épanchement pleural et péritonéale. En 1902, cette association des épanchements était rapportée avec les tumeurs solides de l'ovaire, les myomes du ligament large et les fibromes utérins. En 1904, première thèse de médecine faite par Codet-Boisse, élève de Demons, rapportant une série de 16 cas, insiste sur l'association d'un fibrome ovarien à des épanchements pleural et abdominal. En 1954, Meigs rapporte une série de 84 cas de fibromes de l'ovaire. Un peu plus tard, Funk Bruntano, le décrit avec les fibrothécomes, les tumeurs de la granulosa et les tumeurs de Brenner. Par la suite des cas sont rapportés avec toutes les tumeurs de l'appareil génital féminin [2]. Il est exceptionnellement décrit la disparition des épanchements après l'exérèse d'une tumeur pelvienne maligne aussi avant toute chimiothérapie. Ce constat ramène les auteurs à définir une forme particulière du syndrome de Demons-Meigs, le pseudo-Meigs syndrome, une dénomination à part qui regroupe toutes les autres entités (tumeurs malignes sans cellules néoplasiques dans les épanchements, pseudotumeurs, et autres raretés). Weise et al. [3] ont décrit ce syndrome avec des léiomyomes utérins. Sunitha et al [1] l'ont décrit avec des angioléimyomes utérins. D'autres l'ont décrit avec des tumeurs ovariennes malignes primitives notamment des adénocarcinomes ou des carcinomes endométroïdes, ou bien secondaires métastatiques [4]. Elizabeth et al. [5] ont rapporté ce syndrome avec des cancers digestifs notamment colorectaux. Par ailleurs, d'autres auteurs incluent sous le nom du pseudo-Meigs syndrome, toute tumeur bénigne du tractus génital associée à une pleurésie et une ascite, et de ce fait, ils ont exclus toutes tumeurs malignes pelviennes de ce syndrome [6].

La physiopathologie est encore discutée et reste hypothétique. Une théorie mécanique suggère la persistance du canal pleuropéritonéal et le rôle des troncs lymphatiques transdiaphragmatiques dont la compression tumorale entraine l'ascite qui transsude à travers ce canal pour donner la pleurésie [2]. Mais cette théorie n'explique pas les observations décrites du syndrome en cas de tumeurs de petite taille, parfois millimétriques. La théorie hormonale suggère un dérèglement endocrinien d'une tumeur sécrétante les'strogènes à point de départ génital. En effet, des thécomes minimes ou une hyperplasie thécale est retrouvée à l'examen histologique minutieux de toutes les tumeurs ovariennes accompagnant ce syndrome (fibrome, kyste, goitres ovariens, cystadénocarcinome..) ou même lors de certains états physiologiques (ovulation, grossesses, début de ménopause) [7].

Les CA 125 sériques, marqueurs antigéniques de l’épithélium coelomique et de ses dérivés, ne sont pas spécifiques des tumeurs ovariennes. Il est classique au cours de ce syndrome de mettre en évidence une élévation du CA 125 [7], Un taux élevé n'est pas un bon indicateur de malignité ovarienne et peut être en rapport avec la quantité de l’épanchement ascitique, elle-même en rapport avec la taille tumorale [7–11]. Dans notre observation, quoiqu'il s'agisse d'une tumeur maligne, le taux du CA 125 n’était pas très élevé que ce que le voudrait un cancer ovarien métastatique.

L'association d'un cystadénocarcinome ovarien avec une pleurésie et une ascite plaiderait plutôt en faveur de l'origine métastatique de ces épanchements. En revanche, la négativité des ponctions biopsies pleurales, des biopsies péritonéales, épiploïques et ganglionnaires, l'absence de cellules malignes sur les études cytodiagnostiques du liquide pleural et péritonéale et la disparition de ces épanchements après l'exérèse chirurgicale de la tumeur et avant toute chimiothérapie, plaideraient en faveur d'un pseudo-syndrome de Meigs.

## Conclusion

Le pseudo-Meigs syndrome est une entité rare, sa physiopathologie reste obscure. Le critère majeur repose sur l'exclusion de l'origine métastatique des épanchements pleural et péritonéal, et leur disparition après l'exérèse tumorale de la tumeur pelvienne et avant toute chimiothérapie. Une telle distinction est importante aussi bien sur le plan thérapeutique que pronostique.
